# Arsenic trioxide induces autophagic degradation of the FLT3-ITD mutated protein in FLT3-ITD acute myeloid leukemia cells

**DOI:** 10.7150/jca.29751

**Published:** 2020-03-13

**Authors:** Xiao-Jian Liu, Li-Na Wang, Zu-Han Zhang, Cong Liang, Yu Li, Jie-Si Luo, Chun-Jin Peng, Xiao-Li Zhang, Zhi-Yong Ke, Li-Bin Huang, Yan-Lai Tang, Xue-Qun Luo

**Affiliations:** 1 Department of Pediatrics, The First Affiliated Hospital, Sun Yat-sen University, Guangzhou 510080, China; 2 Department of Pediatrics, Guangzhou Women and Children's Medical Center, Guangzhou 510623, China

**Keywords:** FLT3-ITD, acute myeloid leukemia, arsenic trioxide, autophagy

## Abstract

The prognosis of acute myeloid leukemia (AML) with FMS-like tyrosine kinase 3 internal tandem duplication (FLT3-ITD) mutations is poor. Some studies, including our previous study, have indicated that arsenic trioxide (ATO) exhibited significant anti-carcinogenic activity in FLT3-ITD AML cells and explored the possibility of targeting the FLT3-ITD protein for degradation as a therapy. Autophagy is a critical mechanism of the anti-leukemic effects of ATO. In this study, we explored the therapeutic efficacy of ATO treatment in a mouse model bearing FLT3-ITD AML and found that ATO significantly reduced the leukemic burden in bone marrow and spleen. We also found that autophagy was responsible for, at least in part, the degradation of the FLT3-ITD protein by ATO. After ATO treatment, MV4-11 cells showed complete autophagic flux. The autophagy inhibitor bafilomycin A or down-regulation of the key autophagy genes Atg5 and Atg7 reversed the FLT3 degradation induced by ATO. We also found that p62/SQSTM1 delivered FLT3-ITD proteins to the lysosome, where they were subsequently degraded. These results indicate that ATO can induce autophagic degradation of the FLT3-ITD mutated protein in FLT3-ITD AML.

## Introduction

The internal tandem duplication (ITD) mutations of FMS-like tyrosine kinase 3 (FLT3) occur in approximately 25% of acute myeloid leukemia (AML) cases and are associated with poor therapeutic efficacy and short survival 1. Many FLT3-ITD kinase inhibitors have been researched and tested in clinical trials, but the responses are still limited 1. Reports 2-4 have shown that arsenic trioxide (ATO) alone or in combination with all-trans retinoic acid (ATRA) improved prognosis in acute promyelocytic leukemia (APL) with FLT3-ITD. Several studies 5-7, including our previous study 6, demonstrated that ATO exhibited significant anti-carcinogenic activity, promoted the degradation of the FLT3-ITD protein and inhibited its downstream signaling pathways in FLT3-ITD AML cells. However, how ATO promotes the degradation of the FLT3-ITD protein remains unclear.

Autophagy is a critical mechanism of the anti-leukemic effects of ATO and contributes to the ATO-induced degradation of fusion oncoproteins such as PML-RARA in APL cells and BCR-ABL in chronic myelocytic leukemia (CML) cells 8-11. ATO also induces autophagy, which leads to cell death in many human solid tumors, such as osteosarcoma, glioma, prostate cancer, and others 12-14. Autophagy is a highly conserved biological process through which protein aggregates and damaged organelles are sequestered into double-membrane vesicles, called autophagosomes, which subsequently fuse with a lysosome to degrade the cargo 15. Cargo selection was achieved through the autophagy adapter, which cross-links the LC3-modified autophagosome membrane with the ubiquitination target destined for degradation 16. P62/SQSTM1 is a key selective autophagy adapter that plays an important role in the degradation of PML-RARA, BCR-ABL and other oncoproteins during ATO treatment 10,17. Whether ATO induces autophagy in FLT3-ITD AML cells and promotes the degradation of the FLT3-ITD protein still needs to be explored.

## Materials and Methods

### Cells and reagents

The FLT3-ITD-expressing MV4-11 cell line and the drug ATO were described in our previous studies 6. Bafilomycin A1 (Sigma-Aldrich, St Louis, MO, USA) was dissolved in dimethyl sulfoxide (DMSO) as a stock solution at 100 µmol/L and stored at -20°C. Controls and siRNAs were purchased from Guangzhou RiboBio Co., Ltd. (Guangzhou, Guangdong, China).

### Tumor xenografts in NSI mice and cytometric analysis

MV4-11 cells (2×10^6^ cells/mouse) were transplanted by tail-vein injection into irradiated (100 cGy) 4-week-old NOD-SCID-IL2Rg-/- (NSI) mice, which were generated by TALEN-mediated IL2Rg gene targeting in NOD/SCID mice (Guangzhou Institutes of Biomedicine and Health, Chinese Academy of Sciences). Ten days after the injection, mice were randomly split into 2 groups of at least 5 mice and then treated with either the control (PBS, Beyotime, Shanghai, China) or ATO (1.5 mg/kg) twice per day by intravenous injection. Two weeks later, mice were euthanized, and then, bone marrow cells and spleen cells were harvested. The engraftment of MV4-11 cells, labeled with anti-hCD45-PE and anti-hCD 33APC (eBioscience, San Diego, CA, USA), was assessed by flow cytometric cell sorting (BD FACSAria II flow cytometer). Histological sections of bone marrow and spleen from NSI mice were prepared and stained with hematoxylin and eosin (H&E) using standard methods. Cell morphology was examined using light microscopy.

### Co-immunoprecipitation (Co-IP) and immunoblotting

Immunoprecipitation of p62/SQSTM1 was performed using Dynabeads Protein A (Invitrogen, CA, USA) according to the manufacturer's protocol. MV4-11 cells were lysed using a cold cell lysis buffer with a protease inhibitor cocktail (Beyotime, Shanghai, China) on a rotator for 1 h and then centrifuged at 4°C for 10 min. The supernatants were incubated with anti-p62 antibody or IgG and then rotated at 4°C overnight. The protein A-Sepharose suspension was added and rotated at 4°C for 2 h. The supernatants were discarded after the magnetic beads aggregated, and the magnetic beads were washed 3 times with NETN (900 mmol/L NaCl) and then added to the protein lysate and loading buffer. Finally, the samples were analyzed by western blotting. Western blotting was performed as previously described. The samples were separated with a 10% polyacrylamide gel, transferred to a PVDF membrane (Millipore, Beijing, China) and probed with antibodies. Anti-Flt3, anti-phospho-Flt-3 (Y591), anti-Atg5, anti-Atg7, anti-p62/SQSTM1, anti-LC3B, anti-GAPDH, and normal mouse IgG were obtained from Cell Signaling Technologies (Beverly, MA, USA).

### Cell transfection with siRNA and mRFP-GFP lentivirus vector

Cell transfection with siRNA was performed as described previously 6. The lentivirus carrying mRFP-GFP-LC3 was purchased from Hanbio Co., Ltd. (Hanbio, Shanghai, China). MV4-11 cells (2.0×10^5^ cells/well) were seeded in 24-well plates and incubated with the lentivirus at an MOI of 100 for 12 h at 37°C. The cells were then transferred to the new medium and incubated for 48 h. Then, the cells were treated with ATO for 0, 12, and 24 h, and fluorescence signals were observed under a confocal fluorescence microscope (Carl Zeiss, Jena, Germany).

### Immunofluorescence microscopy

Cells were harvested and fixed in 4% formaldehyde for 30 min at room temperature and washed with PBS three times. Then, the cells were permeabilized with 0.1% Triton X-100 for 10 min and blocked for 2 h with 2.5% BSA in PBS. After blocking, the cells were incubated with mouse monoclonal anti-Flt3 (R&D Systems, Minneapolis, MN) and rabbit monoclonal anti-P62 (CST, Beverly, MA, USA) overnight at 4°C, washed 3 times with PBS and incubated with both anti-mouse and anti-rabbit IgG Alexa Fluor-conjugated secondary antibodies (Invitrogen, CA, USA) for 1 h. Fluorescence signals were analyzed by a Carl Zeiss LSM 780 microscope (Carl Zeiss, Jena, Germany). At least 10 cells from each group were analyzed.

### Transmission electron microscopy

Cells were fixed with 3% glutaraldehyde for 4 h at room temperature and washed four times with PBS. Then, the cells were fixed again in 1% osmium tetroxide for 2 h and rinsed thoroughly with distilled water. After gradient dehydration with ethanol, cells were embedded in Epon 812 (SPI Supplies, West Chester, PA, USA), polymerized for 24 h at 60°C and then cut into ultrathin sections. These sections were observed using a JEM1400 electron microscope (JEOL, Akishima, Tokyo, Japan) to quantify the autophagosomes and autophagolysosomes. Images were digitally acquired from a randomly selected pool under each condition. At least 10 cells per group were analyzed.

### Data Sources

In this study, one gene expression dataset of AML patients was downloaded from The Cancer Genome Atlas (TCGA, https://www.cancer.gov/ tcga). TCGA expression profiles of 411AML patients was used to train a predictive signature.

### Statistical analysis

All experiments were performed 3 times, and the results are presented as the mean±SD. Two statistical tests were performed to validate significance: the t-test and one-way analysis of variance (ANOVA). Statistical analyses were performed using GraphPad Prism 5 software. P-values of 0.05 or less were considered statistically significant.

## Results

### ATO treatment reduced the leukemic burden in the major hematopoietic organs of mice

**Figure 1:**
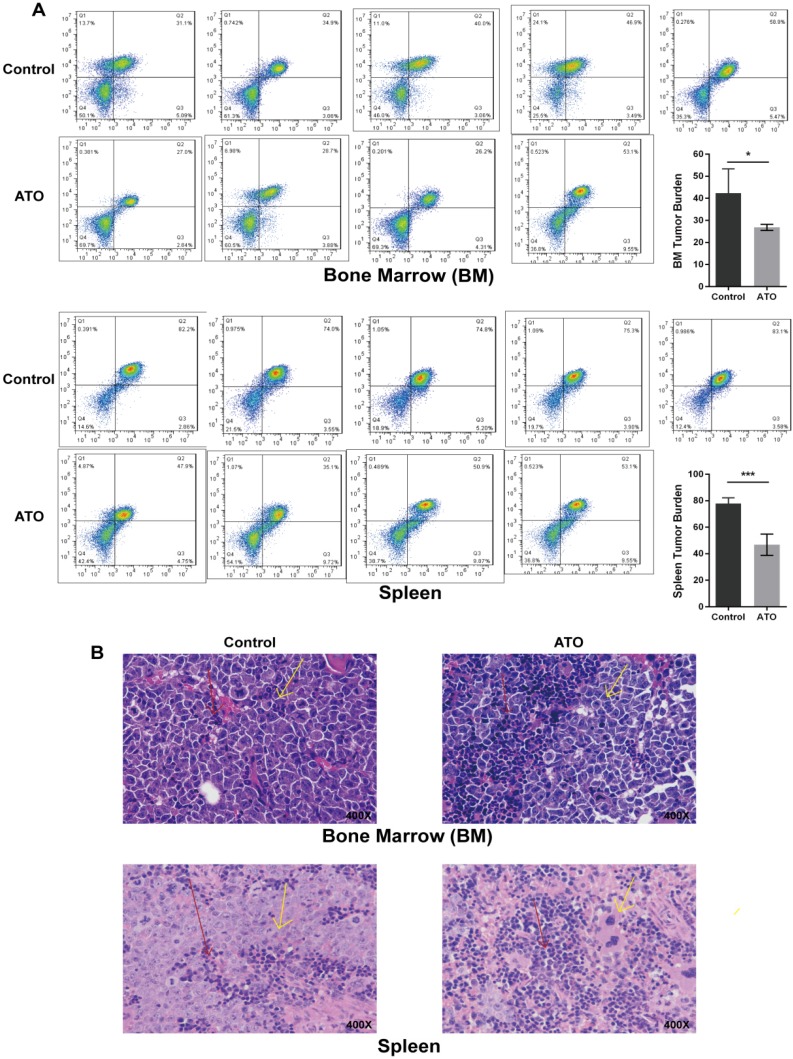
** ATO inhibited tumor growth in NSI mice engrafted by tail-vein injection of MV4-11 cells.** Every mouse received 2×10^6 MV4-11 cells via tail vain injection. Ten days later, mice were randomly split into 2 groups of at least 5 mice and then treated with either control (PBS) or ATO (1.5 mg/kg) twice per day by intravenous injection. Two weeks later, the mice were euthanized, and bone marrow cells and spleen cells were harvested. **(A)** Human leukemia cells in the murine bone marrow and spleen were assessed by hCD45/hCD33 labeling and flow cytometry (* P<0.05; *** P<0.001). **(B)** Histological sections of bone marrow and spleen were prepared and stained with hematoxylin and eosin (H&E). Cell morphology was examined using light microscopy. Red arrows indicate murine cells, and yellow arrows indicate human cells.

Our previous study identified the effect of ATO-induced apoptosis and growth inhibition on FLT3-ITD AML cell lines 6. Next, we explored the effect of ATO on leukemic burden in a xenograft FLT3-ITD AML mouse model using the MV4-11 cell line. *In vivo* dosing of ATO (1.5 mg/kg body weight) was well tolerated in NSI mice. The therapy significantly reduced the tumor burden on day 14 compared with that of mice treated with PBS, as shown by the decreasing bone marrow and spleen human leukemia cells determined by flow cytometry (Figure 1A) and histochemistry (Figure 1B).

### ATO treatment induced autophagy in MV4-11 cells

**Figure 2:**
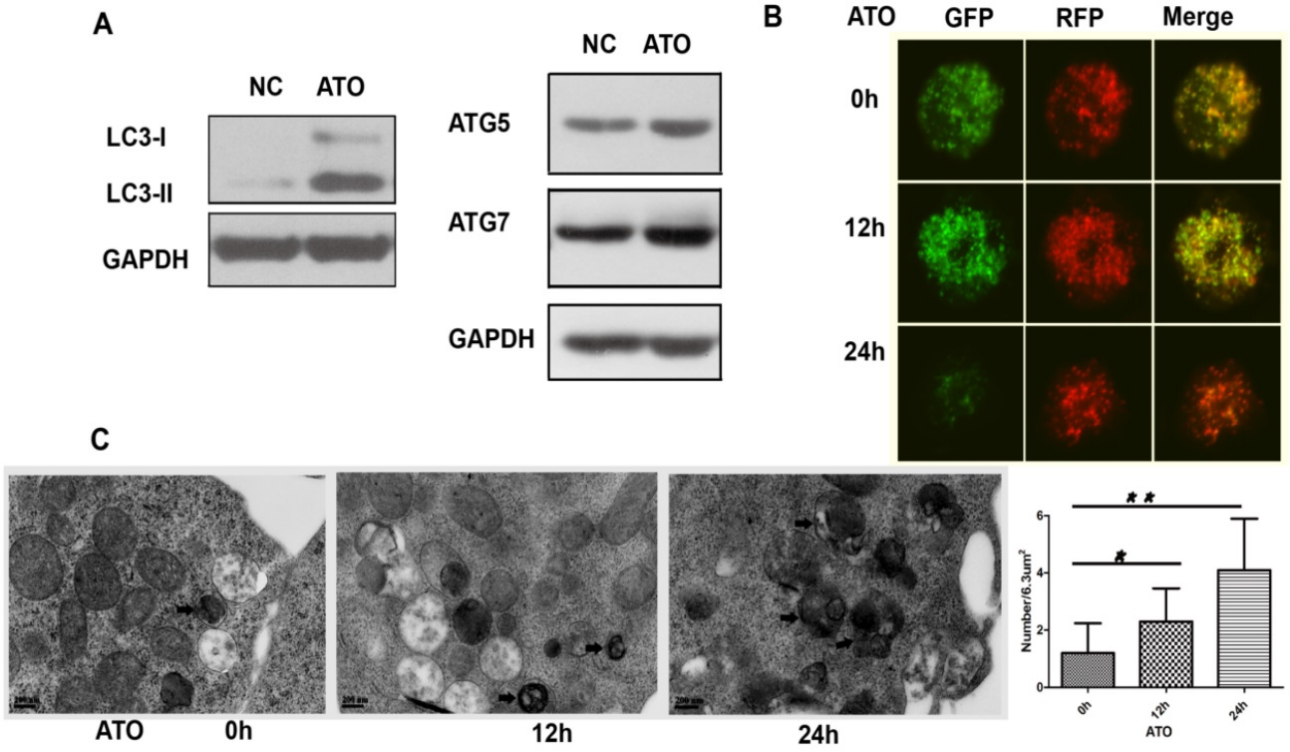
** ATO induced autophagy in the FLT3-ITD AML cell line. (A)** MV4-11 cells were treated with ATO for 24 h, and the expression levels of LC3, ATG5 and ATG7 were detected by western blot analysis. GAPDH expression was used as a loading control. **(B)** MV4-11 cells transfected with mRFP-GFP-LC3 plasmids were treated with ATO for 0, 12, and 24 h and then observed by confocal fluorescence microscopy. Scale bar=5 µm. **(C)** MV4-11 cells were treated with ATO for 0, 12, and 24 h. The number of autophagosomes was observed by transmission electron microscopy and calculated (each group had 30 views, * P<0.05, ** P<0.01). Black arrows represent autophagosomes or autophagolysosomes. Scale bar=2 µm.

**Figure 3:**
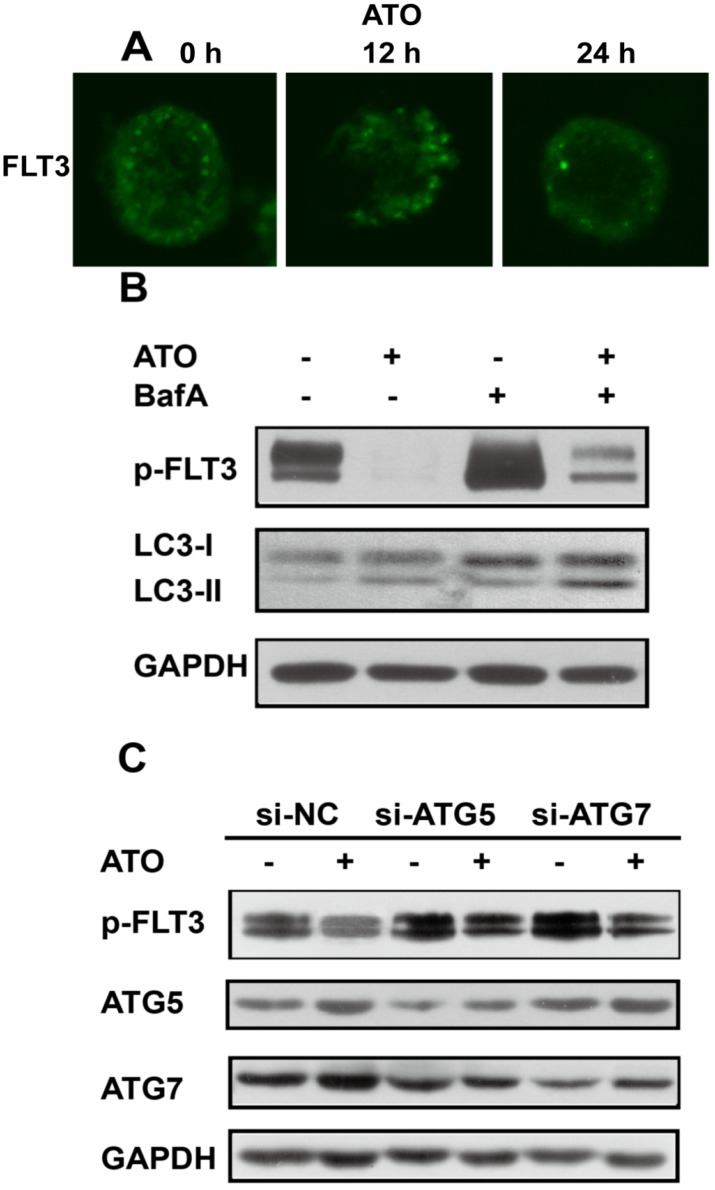
** Degradation of FLT3-ITD by ATO was reversed by inhibition of autophagy (A).** MV4-11 cells were treated with ATO for 0, 12, and 24 h, and immunofluorescence staining of p-FLT3 was analyzed. **(B).** MV4-11 cells were treated with ATO alone or in combination with bafilomycin A (BafA) for 24 h, and p-FLT3 and LC3-I to LC3-II conversion were assessed by western blot analysis.** (C).** MV4-11 cells were transfected with siRNAs targeting Atg5 or Atg7 for 48 h and then treated with or without ATO for 24 h. Then, p-FLT3, ATG5 and ATG7 were assessed by western blotting. GAPDH expression was used as a loading control.

To explore whether autophagy is involved in the cytotoxicity of ATO on FLT3-ITD cells, we examined the processing of full-length LC3-I to LC3-II, an autophagy marker, and the autophagy-related proteins ATG5 and ATG7 in ATO-treated MV4-11 cells. ATO increased the conversion of LC3-I to LC3-II and the protein levels of ATG5 and ATG7 (Figure 2A). Autophagic flux is a dynamic process. To detect autophagic flux, we subsequently transfected the exogenous LC3 plasmids with green (GFP) and red (RFP) fluorescence (mRFP-GFP-LC3) by the lentivirus system and analyzed the fluorescence signals by confocal fluorescence microscopy. In the early stage of autophagy, autophagosomes show dual red and green fluorescence, merging as a yellow fluorescence signal. In the late stage of autophagy, autophagosomal-lysosomal fusion shows only red fluorescence because GFP is sensitive to lysosomal proteolysis and can be quenched in acidic conditions, whereas RFP is not. Our results showed that the accumulated yellow signal increased in MV4-11 cells treated with ATO for 12 h, indicating the formation of autophagosomes. Then, the green fluorescent component of the composite yellow fluorescence was lost after 24 h, indicating that autophagosomes were fused with lysosomes (Figure 2B). Ultrastructural analysis, which was performed by transmission electron microscopy (TEM), revealed that autophagosome formation was induced in MV4-11 cells after ATO was added for 12 h and decreased after 24 h (Figure 3C). Taken together, these results showed that ATO treatment of MV4-11 cells induced a complete autophagic process.

### Degradation of FLT3-ITD by ATO was reversed by inhibition of autophagy

Our previous study showed that ATO could down-regulate FLT3-ITD protein levels, as shown by western blot analysis 6. In order to precise the mechanisms, we first analyzed FLT3 mRNA levels of MV4-11cells treated with ATO and found that it was not changed too much(Supplementary Figure 1A). Then, we blocked the synthesis of new proteins by the protein synthesis inhibitor cycloheximide (CHX) and found that ATO shortened the half-life of FLT3-ITD to a greater extent than DMSO (the control) did (Supplementary Figure 1B). Those results suggest that ATO may regulate the degradation of FLT3-ITD. Next, we investigated the role of ATO in FLT3-ITD expression by immunofluorescence microscopy analysis of MV4-11 cells and further demonstrated that FLT3-ITD could be down-regulated by ATO in a time-dependent manner (Figure 3A). To investigate the role of autophagy in ATO treatment of FLT3-ITD cells, we treated MV4-11 cells with ATO alone or in combination with the autophagy inhibitor bafilomycin A for 24 h. The results showed that the conversion of LC3-I to LC3-II was increased while p-FLT3 was decreased by ATO, but the degradation of FLT3 was reversed by the addition of bafilomycin A (Figure 3B). Furthermore, MV4-11 cells were transfected with siRNAs against Atg5 and Atg7, 2 key autophagy-related genes, and then treated with or without ATO for 24 h. Then, p-FLT3, ATG5 and ATG7 were assessed by western blotting. We confirmed that inhibition of autophagy with siRNA against autophagy-related genes restored FLT3 expression in ATO-treated cells (Figure 3C). Taken together, these results demonstrated that autophagy might be responsible for, at least in part, the degradation of FLT3-ITD during ATO treatment.

### The combination of FLT3 and p62/SQSTM1 occurred via an ATO-dependent mechanism, ultimately resulting in the degradation of FLT3-ITD

**Figure 4:**
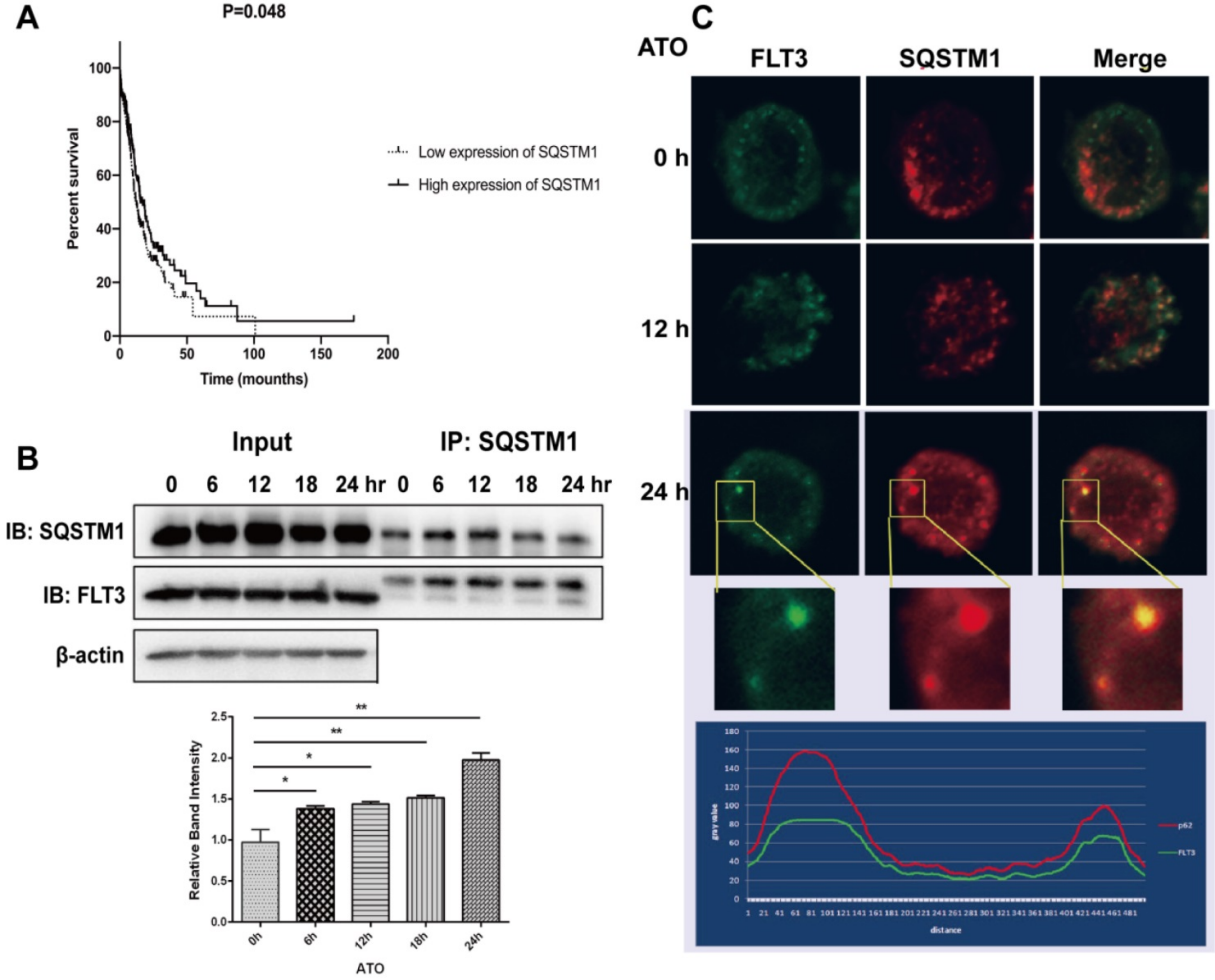
** The combination and colocalization of FLT3 and p62/SQSTM1. (A)** The SQSTM1 expression dataset of AML patients was downloaded from TCGA. **(B)** MV4-11 cells were treated with ATO for 0, 6, 12, 18, and 24 h. Cell lysates were immunoprecipitated (IP) with p62/SQSTM1 antibody or control IgG. IP proteins were assessed by western blot with FLT3 and p62/SQSTM1 antibodies, and β-actin expression was used as a loading control. **(C)** MV4-11 cells were treated with ATO for 0, 12, and 24 h, and then, cells were stained in indirect immunofluorescence analyses by fluorescein-conjugated antibody. Fluorescence signals of FLT3 (green) and p62/SQSTM1 (red) were detected by confocal microscopy. Merged panels indicate overlapping images of the 2 fluorescence signals.

Studies have reported that ATO targets oncoproteins via p62/SQSTM1-mediated localization of oncoproteins to autolysosomes and subsequent degradation 10,17. In this study, we investigated whether p62/SQSTM1 is involved in the potential mechanisms of ATO-induced FLT3-ITD degradation. We examined that the lower expression of SQSTM1 was associated with a poor prognosis of AML patients (Figure 4A). Furthermore, we studied whether FLT3 could directly interact with p62/SQSTM1. MV4-11 cells were treated with ATO for different times ranging from 0 to 24 h, and lysates were immunoprecipitated with an anti-p62/SQSTM1 antibody and immunoblotted with an anti-FLT3 antibody to detect FLT3-ITD. The results showed an ATO-inducible interaction between FLT3 and p62/SQSTM1 that increased in a time-dependent manner (Figure 4B). To further investigate the localization of FLT3 and p62/SQSTM1, we performed immunofluorescence analyses, and the results showed that FLT3 colocalized with p62/SQSTM1 (Figure 4C). These results indicated that ATO physically linked FLT3 to p62/SQSTM1 in autophagosomes and autolysosomes and contributed to the degradation of the FLT3-ITD protein.

## Discussion

FLT3-ITD AML has an adverse prognosis, and mutations are related to an increased tumor burden. In our previous study, we demonstrated that ATO could inhibit growth, promote apoptosis, and down-regulate FLT3 and its downstream signaling pathways in FLT3-ITD AML cells 6. In this study, we explored the therapeutic efficacy of ATO treatment in a mouse model bearing FLT3-ITD AML. Drug-treated mice showed a significant reduction in the tumor burden in bone marrow and spleen.

We also found that autophagy was responsible for, at least in part, the degradation of the FLT3-ITD protein by ATO. After ATO treatment, MV4-11 cells showed a complete autophagic flux. The autophagy inhibitor bafilomycin A or downregulation of the key autophagy genes Atg5 and Atg7 reversed the FLT3 degradation induced by ATO. In addition, we identified an interaction between FLT3 and p62/ SQSTM1 during the treatment of MV4-11 cells with ATO. P62/SQSTM1 is a multifunctional protein possessing multiple domains, including an LC3- interacting region (LIR) and a ubiquitin-associated (UBA) domain, which indicate it can recruit ubiquitinated cargos and bind to LC3, playing a role in exclusive sequestration of ubiquitinated cargo during the formation of the autophagosome. Studies 18 have reported that 19 FLT3 can be ubiquitinated in a certain environment, which further supports the conclusion of our study: p62/SQSTM1 delivers FLT3-ITD proteins to the lysosome, where they are subsequently degraded, which provides a theoretical foundation to support the therapeutic potential of ATO in FLT3-ITD AML.

There are two major protein degradation pathways: the ubiquitin-proteasome pathway and autophagy. Notably, FLT3-ITD can be degraded in both ways. Nishioka et al. reported that the histone deacetylase inhibitor MS-275 induced acetylation of heat shock protein 90 (HSP90) in conjunction with ubiquitination of FLT3, leading to degradation of FLT3-ITD proteins via the ubiquitin/proteasome pathway 20. Oshikawa et al. reported that the Hsp90 inhibitor 17-AAG induced the polyubiquitination and proteasomal degradation of FLT3-ITD 21. Larrue C et al. found that proteasome inhibitor bortezomib- induced autophagy was responsible for the early degradation of FLT3-ITD 22. However, whether ATO can also promote the degradation of the FLT3- ITD protein by the ubiquitin-proteasome pathway remains to be further studied.

## Conclusions

Collectively, in this study, we explored that ATO reduced the leukemic burden in the major hematopoietic organs of mice and induced the autophagic degradation of the FLT3-ITD protein in FLT3-ITD acute myeloid leukemia cells.

## Supplementary Material

Supplementary figures and tables.
