# Supplementary figures and images for: Arsenic trioxide induces autophagic degradation of the FLT3-ITD mutated protein in FLT3-ITD acute myeloid leukemia cells
Source: J Cancer. 2020 Mar 13;11(12):3476–82. doi: 10.7150/jca.29751 (PMC7150460; doi:10.7150/jca.29751)

B

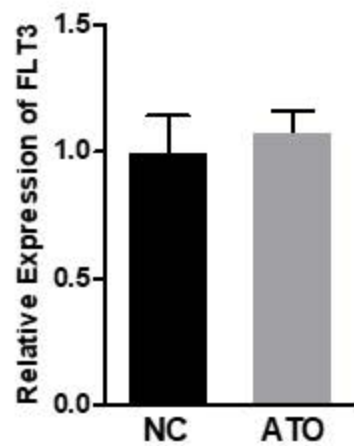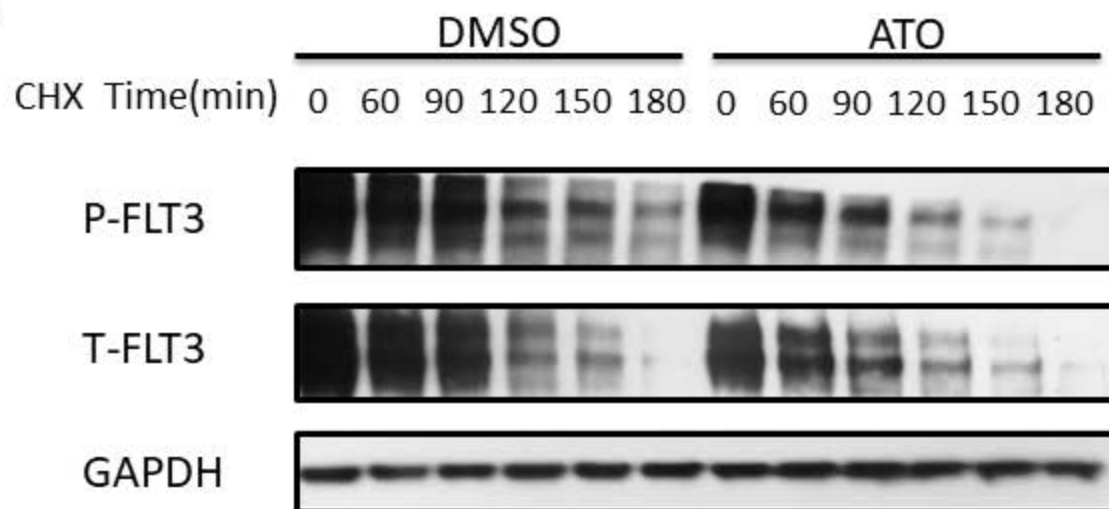

Supplement: Supplementary file 1 — Supplementary figures and tables. [file jcav11p3476s1.pdf]
